# Comparison of ceramic-on-ceramic bearing vs ceramic-on-highly cross-linked polyethylene-bearing surfaces in total hip arthroplasty for avascular necrosis of femoral head: a prospective cohort study with a mid-term follow-up

**DOI:** 10.1186/s13018-019-1410-8

**Published:** 2019-11-27

**Authors:** Bin Feng, Yi Ren, Shiliang Cao, Jin Lin, Jin Jin, Wenwei Qian, Xisheng Weng

**Affiliations:** 1 Department of Orthopaedic Surgery, Peking Union Medical College Hospital, Peking Union Medical College, Beijing, 100730 China; 2 Peking Union Medical College, Beijing, 100730 China

**Keywords:** Total hip arthroplasty, Avascular necrosis, Bearing surfaces, Wear rate, Health-related quality of life

## Abstract

**Background:**

The ideal bearing surface for patients of avascular necrosis (AVN) undergoing total hip arthroplasty (THA) remains controversial. The purpose of this study is to evaluate the clinical outcomes, health-related quality of life (HRQL), and wear of the bearing surface between ceramic-on-ceramic (CoC) and ceramic-on-highly cross-linked polyethylene (CoXPE) THA for patients of AVN after midterm follow-up.

**Methods:**

We performed a retrospective case-control analysis of 93 CoC and 77 CoXPE consecutive THAs for patients of AVN. The cases were followed at a minimum 5 years follow-up (average 7 years). Harris hip score (HHS) score and bearing-related complications were assessed. The health-related quality of life (HRQL) was assessed with the Short Form 36 (SF-36). Plain radiographs and computed tomography (CT) were used for radiographic evaluation.

**Results:**

Both the CoC group and CoXPE group showed statistically significant improvements in HHS scores with no difference between the two bearing surfaces. There was no significant difference as for SF-36 at the latest follow-up between two groups, except for significant higher scores in the dimensions of general health in the CoC group (75.7 vs 64.7, *P* = 0.032). No radiographic evidence of osteolysis and loosening was present at the final follow-up. The mean wear rate of the CoC was 0.0096 mm/year and the CoXPE was 0.047 mm/year after evaluation with reconstructed CT.

**Conclusions:**

CoC THAs acts as well as CoXPE THAs for patients with femoral head avascular necrosis after midterm follow-up. CoC bearing can significantly decrease the wearing rate than CoXPE bearing.

## Background

Total hip arthroplasty (THA) has been considered a successful solution for disabling hip conditions after end-stage avascular necrosis of the femoral head (AVNF). While the number of overall total joint arthroplasties continues to increase, the greatest rise is projected to be in the young patient population, with 52% of all joint replacements predicted to be in patients younger than 65 years old [1]. Major limitation affecting THA survivorship in younger patient has been polyethylene (PE) wear and particle-induced osteolysis resulting in aseptic loosening and late failure of the implant [2]. Traditional metal-on-polyethylene (MoP) has the risk of polyethylene wear and mechanically activated corrosion which many increase the risk of revision [3]. Ceramic-on-ceramic (CoC) has the advantage of low levels of wear and is a popular choice of bearing surface for younger patients [4]. Nevertheless, CoC has its own recognized complication risks (insertional and delayed fracture [5, 6] and squeaking [7, 8]). A ceramic-on-highly cross-linked polyethylene (CoXPE) articulation may decrease long-term wear [9] and potentially have a longer survivorship with less ceramic fracture and squeaking than CoC-bearing surface and is increased use in the younger population (less than 65 years old) over the last 5 years as demonstrated in the National Joint Registry [10]. The previous study has reported the comparative result between CoC and CoP [11], but the study focused on conventional polyethylene. There is limited data on the comparative result between CoC and CoXPE. Similarly, health-related quality of life (HRQL) after THA has been reported only in a handful of studies [12, 13]. In this retrospective study, we aim to evaluate the clinical outcomes, HRQL, complications, and wear rate of the bearing surface between CoC and CoXPE for THA patients with AVNF with a minimum follow-up of 5 years.

## Methods

### Patients and surgical information

Between January 2009 and December 2012, we performed 140 patients (178 THAs) consecutive primary cementless THAs with CoC or CoXPE for patients with AVNF. The choice of the bearing surface was according to the senior surgeons’ preference. Informed consent was obtained from all patients, and the study was approved by the ethics committee of our hospital. The patients were prospectively followed. Of these, 6 patients (7 THAs) were lost to follow-up and 1 died. The follow-up was concluded in December 2017. In total, 133 patients (170 THAs) were followed with a minimum follow-up of 5 years. The mean follow-up was 7 years (range 5–9 years). Demographic data are presented in Table 1. There is no significant difference between the groups with regard to the distribution of sex and body mass index (BMI). However, there were younger patients in the CoC group (51 years vs 59 years, *P* = 0.01).

**Table 1:** Demographics of the avascular necrosis patients undergoing primary total hip arthroplasty with CoC or CoXPE bearing with more than 5 years follow-up

	CoXPE (*n* = 77)	CoC (*n* = 93)	*P* value
Patients	62	71	
THAs	77	93	
Age (years)	59 (36–79)	51 (30–75)	*P* = 0.034
Sex (male/female)	44/33	53/40	*P* = 0.984
BMI (kg/m2)	23.2 (19.7–28.9)	25.2 (19.9–29.3)	*P* = 0.162
Cause of AVNF			
Steroids	57	72	
Alcohol	12	15	
traumatic	8	6	
Follow-up (years)	7.2 (5–9)	6.9 (5–9)	

*CoXPE* Ceramic-on-highly cross-linked polyethylene, *CoC* ceramic on ceramic, *BMI* body mass index, *AVNF* avascular necrosis of femoral head. All values were given as the mean and range

All operations were performed through a posterolateral approach in a lateral position. A BIOLOX delta (BIOLOX Delta; CeramTec, Plochingen, Germany) ceramic-on-ceramic bearing was used for the CoC group. Uncemented femoral component (Corail; DePuy, Warsaw, IN) with an uncemented acetabular component (Pinnacle; DePuy, Warsaw, IN) was used. A BIOLOX delta ceramic on highly cross-linked polyethylene was used for the CoXPE group. The socket was fixed with a target positioning of 20° of anteversion and 45° of inclination.

### Clinical analysis

The patients were encouraged to walk on the second postoperative day with the assistance of a crutch. Routine follow-up visits were scheduled for 6 weeks, 3 and 12 months, and then annually thereafter. The clinical outcome was assessed using the Harris hip score (HHS). HRQL was assessed using the Short Form 36 (SF-36). The patients were asked whether any noise had occurred and the type of noise (clicking, squeaking, or other noise). The postoperative complications were recorded, including loosening, ceramic fracture, dislocation, infection, periprosthetic fracture, and reoperation.

### Radiographic analysis

Radiographic outcomes were obtained in the standard anteroposterior (AP) view and in the frog position. The loosening of the component was defined according to Kim’s and Sutherland’s studies [14, 15]. Osteolysis was defined as areas of endosteal, intracortical, or cancellous bone destruction of > 2 mm that were non-linear and were progressive [16].

**Fig. 1:**
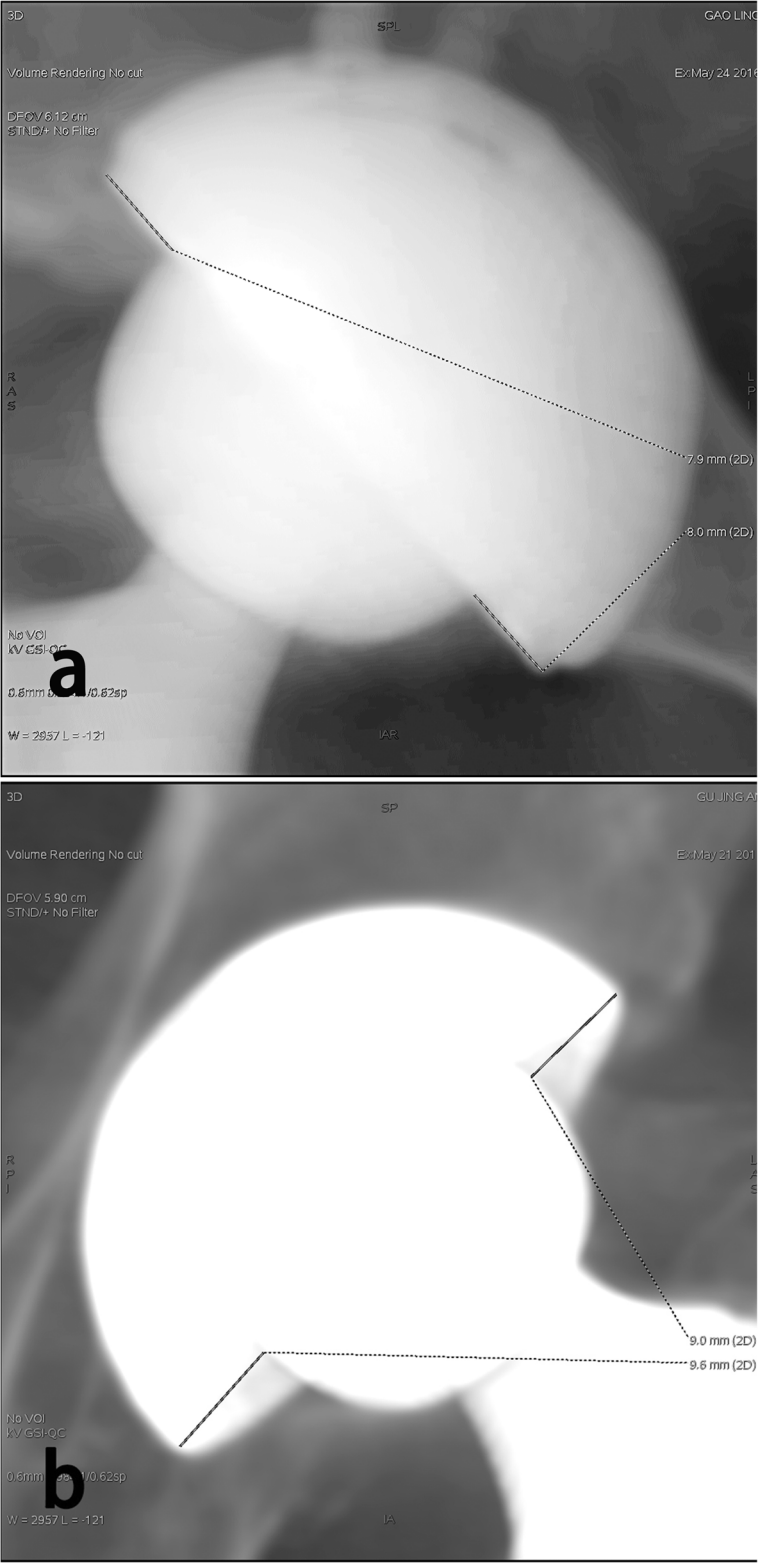
Measurement of femoral head penetration into the liners (measured on reconstructed CT). One line is drawn from the superior to the inferior edge of the acetabular component. The distances from the superior margin of the acetabular component to the femoral head and from the inferior margin of the acetabular component to the femoral head were measured. **a** A 37/F patient underwent THA with CoC bearing for right AVNF, the femoral head penetration was measured on reconstructed CT at postoperative 7 years. **b** A 69/F patient underwent THA with CoXPE bearing for left AVNF; the femoral head penetration was measured on reconstructed CT at postoperative 8 years

The wear of the liner was measured by using a computed tomography (CT) [17]. Artifact subtract reconstructed CT was performed at the latest follow-up. The radiography was measured by two independent surgeons. The annual wear rate was calculated by dividing total femoral head penetration at the end-point of observation by the number of years of follow-up [18] (Fig. 1).

### Statistical analysis

Continuous variables were summarized using means and standard deviation (SD), and categorical variables were summarized using counts and proportions. The level of statistical significance was set at *P* < 0.05. A two-way analysis of variance was used to analyze the difference between the CoC and CoXPE groups for functional outcomes and wear rates. For categorical variables, chi-square analysis was used. All statistical analyses were performed using SPSS 15.0 (SPSS, Inc., Chicago, IL).

## Results

### Clinical outcomes

The mean Harris hip score (HHS) improved from 47.9 points preoperatively to 89.6 points at the final follow-up for CoC group (*P* < 0.01). The mean HHS improved from 40.4 points preoperatively to 86.7 points at the final follow-up for the CoXPE group (*P* < 0.01). There was no significant difference in the HHS at the final follow-up between the group of CoC and CoXPE (*P* = 0.247).

Health-related quality of life (HRQL) was evaluated with Short-Form Health Survey (SF-36) [19] (Table 2). The patients in CoC group showed significant higher scores in general health than CoXPE group (75.7 vs 64.7, *P* = 0.032). There was no significant difference between the two groups as for the other dimensions of SF-36. (Table 2).

**Table 2:** Scores for the dimensions of the SF-36 at the latest follow-up between CoXPE group and CoC group

	CoXPE		CoC		*T* value	*P*
	Mean	SD	Mean	SD		
SF-36						
Physical function	77.5	16.1	83.6	16.2	− 1.4	0.167
Role limitation, physical problem	11.4	12.7	10.3	13.9	0.29	0.773
Role limitation, emotional problem	69.7	20.3	76.7	26.4	− 0.764	0.448
Social function	76.7	23.6	77.2	15.8	− 0.095	0.924
Bodily pain	86.4	17.8	85.0	18.6	0.276	0.784
Vitality	72.7	10.4	75.8	3.8	− 1.613	0.113
Mental health	77.1	11.2	79.8	5.0	− 1.277	0.207
General health	64.7	21.7	75.5	14.9	− 2.198	0.032
Reported health transition	48.8	9.37	48.5	8.6	0.137	0.891

*CoXPE* Ceramic-on-highly cross-linked polyethylene, *CoC* ceramic on ceramic, *SD* standard deviation

### Radiological outcomes

All the patients completed the plain radiograph follow-up. We detected no radiographic evidence of osteolysis and loosening at the final follow-up. One hundred two cases (60%) completed the CT follow-up (57 cases for CoC, 45 cases for CoXPE). The minimal resolution of CT measurement was 0.1 mm in our system. The mean annual liner wear rate was 0.0096 ± 0.003 mm/year for the CoC group according to the reconstruction CT (Fig. 1a), and the mean annual liner wear rate was 0.047 ± 0.009 mm/year for the CoXPE group (Fig. 1b). The CoXPE group had a significantly higher annual wear rate than the CoC group (*P* < 0.001).

### Complications

Three hip dislocations occurred in the CoXPE group (with a ratio of 3.9%). Two occurred within postoperative 1 year, and one occurred at the 8 years after index operation. Six hip dislocation occurred in the CoC group (6.5%). Five occurred within postoperative 1 year, and one occurred at the 6.5 years after index operation. All dislocations were successfully treated conservatively, using a single closed reduction with no recurrence. There was no statistical difference as to the complication of dislocation between the CoC and CoXPE groups (6.5% vs 3.9%, *P* = 0.459) (Table 3). One hip had periprosthetic fracture at the distal part of the stem in the CoXPE group after falling and was successfully treated with open reduction and internal fixation. One hip had a superficial infection in the CoXPE group and was successfully treated with debridement. Two hip reported the noise of “snap” when rising from a squatting position in the group of CoXPE. Eight hips reported postoperative noise in the CoC group, with two for the noise of “grind” and six for the noise of “snap.” These noises were not associated with pain or limitation of function. No patients reported squeaking postoperatively in our group. No patients required revision for the noise. There was a higher rate of the compilation of noise for the group of CoC than the CoXPE (8.6% vs 2.6%, *P* = 0.098) (Table 3). There was no occurrence of ceramic fracture among the CoC group at the latest follow-up. There were no failures or loss of fixation related to bearing surfaces/wear in both groups.

**Table 3:** Comparison of postoperative complication between CoXPE and CoC for avascular necrosis patients underwent primary THA

	CoXPE	CoC	χ^2^	*P* values
Case number	77	93		
Dislocation	3	6	0.549	0.459
Periprosthetic fracture	1	N/A	N/A	N/A
Noise	2	8	2.743	0.098
Superficial infection	1	N/A	N/A	N/A

*THA* total hip arthroplasty, *CoXPE* ceramic-on-highly cross-linked polyethylene, *CoC* ceramic on ceramic, *N/A* not available

## Discussion

Although joint registries demonstrate excellent survival using polyethylene [10]. The incidence of osteolysis in conventional polyethylene was almost 18% [20]. Concerns about wear and osteolysis with polyethylene have led to an increased focus on the use of hard-on-hard articulations. There is a trend for the hard-on-hard bearing surface transition from metal-on-metal to CoC articulation [10]. Ceramics offer the best wear resistance, wettability, scratch resistance, and scratch profile [21]. CoC had a lower incidence of osteolysis than metal-on-metal in primary THAs [22]. CoC bearing was considered to be the ideal bearing surface in the younger, active patient with increased use in younger patients [23]. However, ceramic articulations have the drawbacks of fracture, squeaking, and the risk of acetabular torque [11]. Moreover, head and liner options are limited, such as the lack of a lip liner to improve the stability of the hip. Highly crosslinked-polyethylene also demonstrated good wear resistance [9, 24]. The new highly cross-linked polyethylene inserts may reduce the risks associated with ceramic implants while retaining their longevity with less wear than conventional poly [25]. According to literature, both CoC and ceramic-on-plastic bearing showed better implant survival compared with the metal-on-plastic bearing [13].

In this study, there were no failures of bearing surfaces in either the CoC or CoXPE group in AVN patients who underwent primary THA at an average 7 years follow-up. CoC THAs demonstrated good wear resistance within a 5–9-year follow-up. Participants reported significant improvements in the postoperative Harris hip score. This study also demonstrates that with an average 7 years follow-up, there is no difference in patient-reported outcome measures with SF-36 except for the sub-item of general health, suggesting that the CoC performs just as well as the CoXPE. The difference in general health may be related to the elder age of the CoXPE group.

There are many comparative studies that have examined the outcome comparing CoC-bearing surface to ceramic-on-poly surfaces [11, 12, 26, 27]. Atrey et al. reported comparable survivorship and function after 15 years follow-up between CoC and ceramic-on-conventional polyethylene [11]. The hard-on-soft articulation had higher mean annual wear than hard-on-hard articulation. The author concluded that polyethylene wear and osteolysis may represent issues in the future [11]. The limitation was the cross-linked polyethylene was not adopted and the BIOLOX delta ceramic was not widely available. Beaupre et al. reported CoC and ceramic-on-crossfire-polyethylene had a similar health-related quality of life after postoperative 10 years follow-up. There were no failures of loss of fixation related to the bearing surface in either group. The study was limited to radiographic review with plain radiographs and no data of annual wear rate [12]. In those that had a radiographic follow-up at 10 years (*n* = 57; 66%), no osteolysis was noted in either group [12]. The incidence of osteolysis in both bearings was lower than the 18% rate reported for conventional polyethylene in literature [20]. The annual wear rate in this study was similar to the wear rate in literature [11, 28, 29].

The annual wear rate in the current study of the CoC group was 0.0096 mm/year, supporting the excellent behavior and wear resistance of the ceramic bearing [25]. The annual wear rate of CoXPE in the current study was lower than the result of ceramic-on-conventional poly in literature [11]. The result also supported the good wear resistance of highly crosslinked-polyethylene [24]. Although the mean annual wear in the CoXPE group was significantly higher than the CoC group (0.047 vs 0.011 mm), the osteolysis is uncommon with a wear rate of < 0.1 mm/year according to the literature [30]. As a result, the current study shows that there was no difference of HHS, SF-36 scores and osteolysis between the two groups, suggesting that the CoC performed as well as the CoXPE.

Our study has strengths including the aim to study on the same disease and use more accurate CT data to measure the wear rate and radiological outcome. As we have known, the clinical outcome and implant survival tend to differ based on primary diagnosis [13]. Swarup et al. reported that patient diagnosis is predictive of implant survival with juvenile inflammatory arthritis patients having the lowest implant survival [13]. This study focused on THA for osteonecrosis which is one of the common etiologies for THA in our country. The study can more accurately discover the difference of outcome owing to the different bearing surface selection. CT offers a three-dimensional assessment of the liner wear in contrast to the conventional radiographic examination which may underestimate polyethylene wear. It was reported that CT can detect osteolysis and wear earlier than with conventional radiographic examination before loosening occurs [31].

The reported rate of squeaking and other noises for CoC bearing ranged from < 1 to 21%, and the etiology is multifactorial [32]. In our study, audible noise occurred in 8.6% in the CoC group and 2.6% in the CoXPE group. Although the difference was not statistically significant, there was a higher rate of audible noise for the CoC-bearing surface than the CoXPE-bearing surface. We found no reported postoperative squeaking in our study and no difference in postoperative Harris hip scores between the noise and silent hip groups. Furthermore, no revision was required for the noise.

However, there are some limitations to our study. Firstly, this study is limited by its nature of observation study with a relatively small number of cases. However, we conducted a power analysis to determine the sample size to detect the difference of wear between the 2 groups and found that 52 cases were needed in each group. Additionally, the analysis was based on consecutive cases, with no randomization. In this study, patients who received a CoC THA were younger than those who received a CoXPE THA. This might have been caused by selection bias and may have influenced the clinical outcome results. Secondly, our study is a midterm follow-up result. Longer-term studies are needed to determine the wear rate and incidence of osteolysis for implant longevity between the two groups. Thirdly, we did not obtain a full clinical and radiographic follow-up for all of the respondents. Fourthly, we used different sized femoral heads; this may have influenced the complication of postoperative dislocation and clinical results. Finally, the ceramic insert would cost more than poly. In the current study, no cost-effective analysis has been studied, which is also important for healthcare decision-making [12].

## Conclusion

In summary, our results showed other than greater wear in the cross-link polyethylene group, there was no significant difference in outcome between the CoC and CoXPE THA for osteonecrosis after midterm follow-up in terms of functional outcomes, bearing surface-related complication, radiographic results, and HRQL outcome. Both CoC and CoXPE bearings can behave excellently. As for the longevity of the two bearing surfacing, long-term follow-up study will be needed.

## Data Availability

All data presented in this study are available from the corresponding author upon reasonable request.

## Abbreviations

AP: Anteroposterior
AVN: Avascular necrosis
BMI: Body mass index
CoC: Ceramic-on-ceramic
CoXPE: Ceramic-on-highly cross-linked polyethylene
CT: Computed tomography
HHS: Harris hip score
HRQL: Health-related quality of life
PE: Polyethylene
SF-36: Short Form 36
THA: Total hip arthroplasty
